# Outbreak of Diarrheal Illness Caused by *Shigella flexneri* — American Samoa, May–June 2014

**Published:** 2015-01-16

**Authors:** Julia E. Painter, Allison Taylor Walker, Jarratt Pytell, Motusa Tuileama Nua, Siitia Soliai-Lemusu, Eric Mintz, Ibne Ali, Michele Parsons, Haley Martin, Michael Beach, Anna Bowen, Jennifer Cope

**Affiliations:** 1 Epidemic Intelligence Service, CDC; 2 Division of Foodborne, Waterborne, and Environmental Diseases, National Center for Emerging and Zoonotic Infectious Diseases, CDC; 3 American Samoa Department of Health

On May 9, 2014, a physician at hospital A in American Samoa noticed an abnormally high number of children presenting to the emergency department with bloody diarrhea. Based on preliminary testing of stool specimens, *Entamoeba histolytica* infection was suspected as a possible cause. *Shigella* was also suspected in a subset of samples. On May 22, the American Samoa Department of Health requested assistance from CDC with the outbreak investigation. The goals of the investigation were to establish the presence of an outbreak, characterize its epidemiology and etiology, and recommend control measures. The CDC field team reviewed the emergency department log book for cases of diarrheal illness during April 15–June 13, 2014. During this period, 280 cases of diarrheal illness were recorded, with a peak occurring on May 10. Twice as many cases occurred during this period in 2014 compared with the same period in 2011, the most recent year for which comparable surveillance data were available. Cases were widely distributed across the island. The highest number of cases occurred in children aged 0–9 years. Across age groups, cases were similarly distributed among males and females. These patterns are not consistent with the epidemiology of disease caused by *E. histolytica*, which tends to cause more cases in males of all ages.

Hypothesis-generating interviews with families of 13 patients did not reveal any common water, food, sewage, or event exposures. Eight participants reported having ill household contacts, with family contacts often becoming ill within 1–3 days after the participant’s illness onset. Six stool specimens were sent to CDC. All were negative for ameba, including *E. histolytica*, by multiple laboratory methods. All six specimens were also negative for *Cryptosporidium* and *Giardia* by a polymerase chain reaction test. However, an invasion plasmid antigen H (ipaH) gene sequence, a genetic marker of *Shigella*, was identified in four specimens. Additionally, seven *Shigella* isolates sent to the Hawaii Department of Health and CDC were identified as *Shigella flexneri* serotype 7 (proposed; also referred to as provisional 88-893 or 1c), and five shared an indistinguishable pulsed-field gel electrophoresis pattern.

*Shigella* causes an estimated 500,000 cases of shigellosis per year in the United States (1). Most persons infected with *Shigella* develop diarrhea (sometimes bloody), fever, and stomach cramps 1–2 days after they are exposed to the bacteria. The illness usually resolves in 5–7 days. Careful and frequent hand washing and strict adherence to standard food and water safety precautions are the best defense against shigellosis (2).

Together, epidemiologic and laboratory data suggest this was a shigellosis outbreak with person-to-person transmission. This investigation highlights the importance of building epidemiologic and laboratory capacity for enteric illnesses and enhancing basic hand hygiene and prevention strategies in U.S. territories.
